# Topological Defects in Carbon Matrix are Efficiently Constructed by Joule Thermal Shock for Catalyzing the Growth of Carbon Nanotubes

**DOI:** 10.1002/advs.202520422

**Published:** 2026-01-09

**Authors:** Bin Wang, Shizhen Dong, Guoan Xie, Yanling Yu, Nuoxin Wang, Huakun Liu, Xian Jian, Jinyang Li, Zuowan Zhou, Jianhui Qiu

**Affiliations:** ^1^ School of Materials Science and Engineering Xihua University Chengdu Sichuan P. R. China; ^2^ School of Chemistry Southwest Jiaotong University Chengdu Sichuan P. R. China; ^3^ School of Chemistry and Chemistry Engineering Harbin Institute of Technology Harbin Heilongjiang P. R. China; ^4^ Key Laboratory of Cell Engineering of Guizhou Province Affiliated Hospital of Zunyi Medical University Zunyi Guizhou P. R. China; ^5^ Institute of Energy Materials Science (IEMS) University of Shanghai for Science and Technology Shanghai P. R. China; ^6^ School of Materials and Energy University of Electronic Science and Technology of China Chengdu P. R. China

**Keywords:** growth mechanism of CNTs, joule thermal shock preparation, special carbon catalyzes CNTs growth, Topological defect carbon

## Abstract

This study aims to establish a simple and efficient method for preparing topological defect carbon (TDC) and reveal its catalytic mechanism for the growth of carbon nanotubes (CNTs). TDC is prepared using N‐doped carbon as a precursor through multiple Joule thermal shock treatments, and the efficiency of this method in constructing topological defects in the carbon matrix is confirmed by multi‐dimensional characterization. Subsequently, the TDC catalyzed the growth of CNTs, and a possible catalytic growth mechanism based on the synergistic interaction of multiple defects is proposed. The mechanism proposes that the carbon source molecules are activated through electron transfer from pentagonal topological defects and the slight assistance of other defects, and then the activated molecules form a metastable carbon layer by self‐assembling, combined with dehydrogenation rearrangement according to the defect curvature, which further self‐assembles to achieve the growth of CNTs based on the edge activity of the carbon layer. Therefore, this study not only brings new perspectives to clarify the growth mechanism of specialty carbon‐catalyzed CNTs, but also provides an efficient research and development platform for this type of specialty carbon catalysts.

## Graphical abstract

The Joule thermal shock technique ensures the efficient construction of topological defects in the carbon matrix because the instantaneous cooling suppresses the graphitization rearrangement of the heteroatom removal sites. This topological defect carbon can activate carbon source molecules by electron transfer, and further grow into carbon nanotubes based on the self‐assembly process under defect induction.

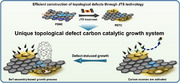

## 1 Introduction

Carbon nanotubes (CNTs) were first discovered by S. lijima in 1991, which set off a research boom on the preparation and application of CNTs [1]. CNTs have a unique coaxial tubular structure of graphite layers, which brings them excellent properties such as high strength, electrical conductivity, and thermal conductivity, and they have been widely used in lithium‐ion batteries, aerospace, electronic devices, and other fields [2, 3]. Currently, CNTs are mainly produced through chemical vapor deposition (CVD) based on metal catalysts (Fe, Co, and Ni), as this technology is low‐cost and easy to industrialize [4, 5, 6]. For CVD technology, metal catalyst will remain in the CNTs because they are coated with carbon, and strong removal of them using strong acids or bases will destroy the structure of CNTs, which significantly limits the subsequent application of CNTs [7, 8]. For example, CNTs are used as conductive agents in lithium‐ion batteries, and their residual metal catalysts can trigger the formation of lithium dendrites, which greatly degrade battery performance [9]. Therefore, how to greatly reduce the impact of catalyst residues on subsequent applications while ensuring that the structure of CNTs is not destroyed is an urgent problem to be solved in the preparation of CNTs using CVD technology.

Some researchers are considering solving the above problems by developing non‐metallic catalysts. The non‐metallic catalysts that have been reported mainly include semiconductor catalysts (Si, SiC), nanoparticle oxide catalysts (SiO_2_, Al_2_O_3_), and special carbon catalysts (diamond, defect‐rich graphite) [10, 11, 12, 13]. For semiconductor catalysts and nanoparticle oxide catalysts, although they are easier to remove than metal catalysts, slight damage to the structure of CNTs is inevitable during the removal process [14]. Regarding special carbon catalysts, some studies believe that even if they remain in CNTs, they will not have a significant impact on the application of CNTs, based on their chemical homology with CNTs (both are mainly composed of CNTs) [15, 16]. Therefore, replacing metal catalysts with special carbon catalysts is considered to be one of the effective ways to solve the problem of metal catalyst residues limiting the application of CNTs. Although the ability of special carbon to catalyze the growth of CNTs has been confirmed, there are still many different opinions about the growth mechanism of CNTs under this type of catalyst. For instance, Jarrn H. Lin et al. concluded that surface defects of special carbon can catalyze the growth of CNTs, and that the growth process is similar to the self‐assembly process of carbon sources on the bulk Au surface [11, 17]. R.P.H. Chang et al. believe that the migration of five‐membered ring and seven‐membered ring defects in the graphite layer is the key to the growth of special carbon‐catalyzed CNTs [18]. However, Yafei Zhang et al. proposed that the edge defects and nano curvature of special carbon are the key factors in the growth of CNTs, and the specific growth process involves the self‐assembly and gradual evolution of nanowires [19]. So far, the following questions remain unresolved: which types of defects are active centers for the growth of special carbon‐catalyzed CNTs, and how the specific growth process proceeds.

Carbon materials inevitably undergo some lattice distortion during the preparation process, and the distortion sites will form non‐hexagonal structures such as five‐membered rings and seven‐membered rings, which are topological defects. These topological defect structures will induce local charge rearrangement of the carbon matrix, which makes the carbon material catalytically active, and the excellent catalytic activity of this type of carbon material has been confirmed in fields such as oxygen reduction reaction and CO_2_ reduction [20, 21]. There is every reason to suspect that this type of topological defect, which is unavoidable and highly active in the carbon matrix, is the active center for the growth of CNTs catalyzed by special carbon. To verify this hypothesis, it is necessary to prepare a carbon material rich in topological defects, namely topological defect carbon (TDC). At present, the preparation of TDC is mainly through the high‐temperature heteroatom removal method (≥ 1100°C). That is, after the heteroatoms in the carbon matrix are removed by high‐temperature treatment, topological defects will form at the corresponding sites [22]. Studies have pointed that this method has the following problems: first, carbon materials are prone to graphitization rearrangement under high‐temperature treatment, which causes the sites to rearrange to form a graphitized structure after the heteroatoms are removed; second, the high‐energy covalent bonds between carbon atoms and heteroatoms are extremely difficult to break by high‐temperature treatment alone, resulting in low efficiency in constructing topological defects [23, 24]. Hence, this method needs new technology or a principle to be improved.

Based on the above research background and conjectures, this study proposes to use the recently emerged Joule thermal shock (JTS) technology [25] to improve the high‐temperature heteroatom removal method in order to achieve the efficient construction of topological defects in the carbon matrix. The improvement strategy is based on the following considerations: i) the instantaneous heating of JTS can provide enough energy to break the covalent bonds between carbon atoms and heteroatoms, while the instantaneous cooling will not give the removal sites enough time to undergo graphitization rearrangement, which will significantly improve the efficiency of topological defect construction; ii) the carbon skeleton undergoes multiple instantaneous heating and cooling oscillations, which may induce additional lattice distortion to form topological defects. Subsequently, in order to verify the hypothesis proposed above, the topologically defect‐rich carbon prepared by multiple JTS treatments was used as a catalyst to catalyze the growth of CNTs through a CVD process. Microstructural characterization shows that multiple JTS treatments can effectively promote the construction of topological defect structures in the carbon matrix; at the same time, catalytic comparison experiments also confirmed the hypothesis of this study, that is, topological defect structures are the key active sites for special carbon to catalyze the growth of CNTs. This study not only brings new perspectives to the mechanism of special carbon catalysis of CNTs growth, but also provides an efficient research and development platform for this type of special carbon catalyst.

## 2 Results and Discussion

**FIGURE 1:**
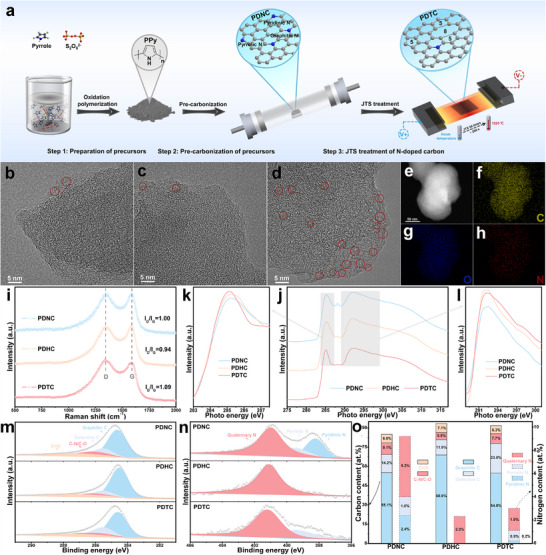
Schematic diagram of the PDTC preparation process based on Joule thermal shock technology (a); HRTEM image of PDNC (b), PDTC (c) and PDHC (d); dark‐field TEM (e), C (f), O (g) and N (h) elemental mapping STEM images of PDTC; Raman spectra of PDNC, PDHC and PDTC (i); C K‐edge NEXAFS spectra (j) and their extended views (k, l) of PDNC, PDHC and PDTC; High resolution C 1s and N 1s XPS spectra of PDNC, PDHC and PDTC (m, n); the proportions of each chemical state of C and N elements in PDNC, PDHC and PDTC are based on XPS spectra (o).

The preparation of the topological defect carbon catalyst by the multiple JTS technique is shown in Figure 1a. First, the precursor PPy was prepared by chemical oxidative polymerization (step 1). Second, PPy was pre‐carbonized, and N‐doped carbon (PDNC) was obtained (step 2). Finally, a topological defect carbon catalyst (PDTC) was obtained by multiple JTS treatments of PDNC (step 3). A specific JTS device is shown in Figure S1a, and when the device is running, the heating area emits strong visible light (Figure S1b). At the same time, the temperature curve (Figure S1c) of the device performing 50 thermal shocks shows that as the number of shocks increases, the upper temperature limit of each thermal shock also increases and finally reaches 1225°C. To highlight the advantage of multiple JTS treatments to construct topological defects in the carbon matrix, comparative catalyst PDHC was also prepared by the reported high‐temperature heteroatom removal (HTHR) method at 1200°C [26].

The microscopic morphology of the catalysts at each stage of the above preparation process was observed by SEM (Figure S2), and it can be found that each carbon catalyst inherited the irregular nano‐spherical morphology of PPy. Furthermore, the graphitized structure of each catalyst was investigated using high‐resolution TEM (HRTEM, Figure 1b–d). As expected, PDHC exhibits a large number of graphitized microcrystalline structures (they have been marked with numerous circles in Figure 1d and summarized in Figure S3), while, in contrast, only very few such structures (the very few areas marked with circles in Figure 1c) are observed for PDTC. TEM element mapping shows that each element is evenly distributed in the PDTC matrix (Figure 1e–h), and compared with PDNC (Figure S4), the N content of PDTC is sharply reduced (Table S1). For PDHC, its N content also dropped sharply compared with that of PDNC. Elemental mapping combined with HRTEM analysis suggested that both multiple JTS treatment and HTHR method can achieve the removal of N heteroatoms in the carbon matrix, and predicted that PDTC treated with the former should have abundant topological defects based on its scarce graphite micro‐crystalline structure.

Raman spectroscopy characterization echoes the conclusions of HRTEM analysis (Figure 1i). For the intensity ratio (I_D_/I_G_) of the D band (which is sensitive to non‐six‐membered ring topological defects and heteroatom doping defects in the carbon matrix) to the G band (which originates from the sp^2^ hybridized carbon in the carbon matrix), which describes the degree of defects in the carbon matrix [27], the order of the catalysts is PDTC>PDNC>PDHC. The above results still indicate that, compared with PDHC, the reduced heteroatom doping defects in PDTC are replaced by a large number of topological defects rather than graphitized microcrystalline structures. Subsequently, the topological defect structures of PDTC were further confirmed by near‐edge X‐ray adsorption spectra (NEXAS, Figure 1j–l). The spectra of all samples have three characteristic peaks located at 285.2, 288.2, and 292.2 eV, which correspond to π* C═C, π* C─O/C─N─C π*, and C─C σ* resonances, respectively [28]. By zooming in on the spectra in the range of 283.0–287.5 eV, it can be clearly found that the π* C═C peak intensity of PDTC is significantly greater than that of PDNC and PDHC. This is believed to be due to the large number of topological defects in PDTC, which leads to an increase in the unoccupied states associated with the π* antibonding orbitals [21]. Meanwhile, the enlarged image of the 288.9–301.0 eV range also compares the C─C σ* peak intensity of each catalyst, which is closely related to the hexagonal graphitization layer [26], and that of PDHC is greater than that of PDTC. In addition, it should be noted that the π* C─O/C─N─C π* peak intensity of PDTC and PDHC is both weakened compared with PDNC, which is obviously attributed to the removal of heteroatoms in the carbon matrix. The above results are consistent with the conclusions of Raman spectra analysis, both indicating that when PDTC is prepared by multiple JTS treatment, a large number of topological defects are formed in the carbon matrix while the N heteroatoms are removed; however, when PDHC is prepared by HTHR method, although the N heteroatoms are also removed, a large number of graphitized microcrystalline structures are formed in the carbon matrix. Furthermore, the TGA curves in air (Figure S5) show that the initial decomposition temperature (IDT, the temperature corresponding to a 5% mass loss) of PDTC (400°C) is significantly lower than that of PDHC (451°C). This is mainly owing to the fact that PDTC has a greater abundance of low‐crystallinity carbons compared to PDHC [29], and it is these low‐crystallinity carbons that constitute a large number of topological defect structures. Hence, the TGA analysis indirectly confirms the results of the Raman and NEXAS spectra analyses, and further suggests that the PDTC obtained by multiple JTS treatments has an extremely high defect density.

In order to deeply explore the chemical state changes of each element during the catalyst preparation process, XPS was performed on each catalyst. Consistent with the element mapping results, both multiple JTS treatment and the HTHR method can effectively remove N heteroatoms from the carbon matrix (Figure S6 and Table S1). The XPS fine spectra of C 1s in different catalysts are presented in Figure 1m. The C 1s spectra of all catalysts can be deconvoluted into four peaks centered at ∼284.6, ∼285.5, ∼286.4, and ∼287.7 eV, which are attributed to graphitic C, defective C, C─N/C─O, and C═O, respectively [30]. Meanwhile, the C contents of each chemical state for all catalysts are summarized in Figure 1o. Obviously, the defective C content of PDTC is much higher than that of PDNC and PDHC, which further proves the high efficiency of multiple JTS treatments to construct topological defects in the carbon matrix. The changes in the chemical state of the N element in each catalyst were also analyzed in depth (Figure 1n). For PDNC, its N 1s spectrum was fitted by the three peaks of pyridinic N (∼398.2 eV), pyrrolic N (∼399.6 eV), and quaternary N (∼401.1 eV) [31]. However, the N 1s spectrum of PDHC can only be fitted by the peak of quaternary N, indicating that the low‐bond‐energy pyridinic N and pyrrolic N are completely removed under the HTHR method, while the high‐bond‐energy quaternary N is partially retained. For PDTC, the residual N heteroatom chemical state is still mainly quaternary N, but a small amount of pyridinic N and pyrrolic N is also retained (Figure 1o). This suggests that compared with the HTHR method, the conditions for constructing topological defects through JTS treatment are relatively mild, which results in the retention of a small number of low‐bond‐energy N heteroatoms. This relatively mild treatment condition suppresses the graphitization rearrangement of sites after the removal of N heteroatoms, thereby improving the construction efficiency of topological defects.

Even more interesting, the difference in preparation conditions had a profound impact on the specific surface area (S_BET_) of the catalyst. PDHC has undergone intense and continuous high‐temperature treatment, which causes the microporous structure and amorphous carbon in the carbon matrix to collapse/merging and merge, respectively, [32, 33] thus significantly reducing its S_BET_ (Figure S7 and Table S2). In addition, the high‐temperature treatment caused the light components of the sample to be further decomposed based on the changes in sample mass before and after treatment (sample mass before and after treatment were ∼ 0.50 and ∼ 0.42 g, respectively). However, these components did not form the microporous structure that contributes to S_BET_ in the carbon matrix after decomposition owing to the excessively high processing temperature. On the contrary, the instantaneous heating and cooling process experienced by PDTC did not provide sufficient time and energy for the collapse of the microporous structure and the graphitization of amorphous carbon, but the lightweight components were also decomposed (sample mass before and after treatment were ∼0.50 and ∼ 0.45 g, respectively). Therefore, after these components are decomposed, a large number of microporous structures are formed in the carbon matrix, which significantly increases the S_BET_ of PDTC. Similar results have been reported in reference [34], and the corresponding characterizations also support the above view (Table S2, the V_mic_ of PDTC being 16 times that of PDHC). These changes in the pore structure and S_BET_ of PDTC may have a positive impact on the exposure of its defective active sites.

Although the above characterizations have indirectly confirmed that PDTC based on multiple JTS treatment has abundant topological defects, the specific structure of these defects and their formation process have not yet been revealed. Therefore, a curved graphene model containing three N‐doping forms (pyridinic N, pyrrolic N, and quaternary N) was established (Figure S8a1,b1), and multiple JTS treatments were simulated by ReaxFF MD to analyze the formation process of topological defects. The upper limit of the thermal shock temperature in this ReaxFF MD was set to 4173 K, which is far higher than the actual temperature. This is a common strategy in ReaxFF MD to speed up the reaction in order to keep the simulation within the available time scale [27]. In the early stage of the simulation, it was observed that the bond cleavage of the three types of N doping forms proceeds in the following order: first the pyrrolic N, and then the pyridinic N, and finally the quaternary N (Figure S8 a2–b4, the circled parts). This order exactly matches the bond energy order of the three types of N‐doping forms, indicating the rationality of this simulation. As the simulation continued, large‐area vacancy defects containing dangling bonds were formed in the carbon matrix when the N heteroatoms were removed (Figure S8b5,b6), and these defects were eventually repaired by dangling bond rearrangement to form a large number of topological defects (Figure S8b7,b8). It was also noted that although the residual N heteroatoms in the carbon matrix after the simulation were mainly quaternary N, the amount of residual pyrrolic N was slightly higher than that of pyridinic N, which was consistent with the XPS analysis and once again suggested the reliability of the simulation. This phenomenon can be understood as follows: although the low‐bond energy pyrrolic N is easy to remove, it also means that it is easy to form; therefore, under this dynamic mechanism, its residual amount is ultimately slightly greater than that of pyridinic N.

**FIGURE 2:**
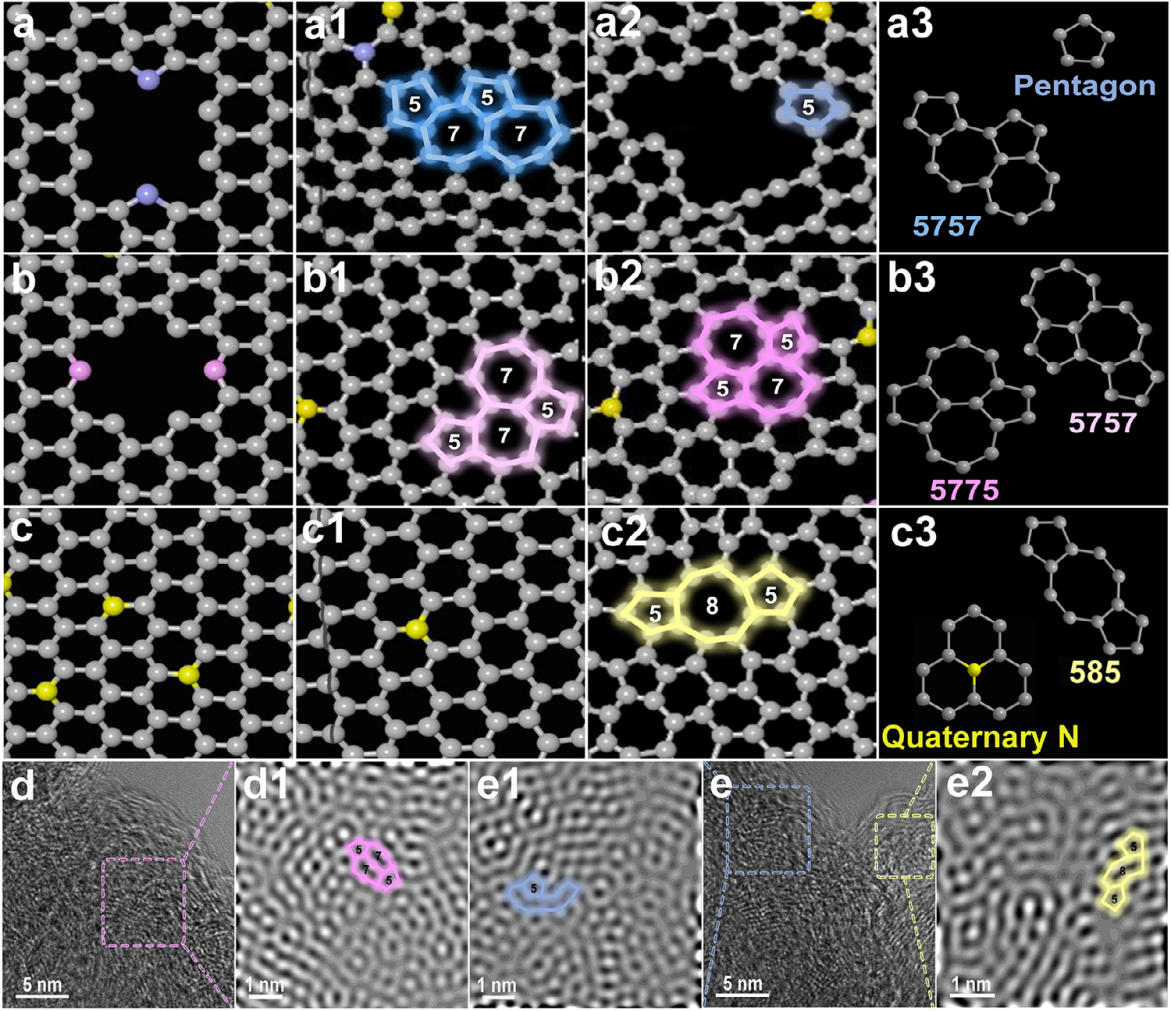
Structural evolution of each N‐doping type based on ReaxFF MD: (a–a3) for pyrrolic N, (b–b3) for pyridinic N, and (c–c3) for quaternary N; ACTEM image of PDTC (d, e) and the magnified images of the corresponding areas after FFT (d1, e1, and e2).

The evolution of each N‐doped structure was tracked based on ReaxFF MD simulation, and the evolved structures were extracted (Figure 2). For pyrrolic N, they mainly evolve into pentagonal and 5757 topological defects after being removed (Figure 2a–a3). Similar to pyrrolic N, pyridinic N is mainly transformed into 5757 and 5775 topological defects (Figure 2b–b3). For quaternary N, some of the removed ones are converted into 585 topological defects, while some remain unchanged (Figure 2c–c3). Summarizing the evolution rules of N‐doped structures, pyrrolic N and pyridinic N mainly evolve into topological defects with alternating five‐membered rings and seven‐membered rings, while only quaternary N can evolve into topological defects containing eight‐membered rings. In order to visually observe the topological defects in the carbon matrix, PDTC was characterized by ACTEM (Figure 2d,e), and the results showed that there were abundant topological defects in the PDTC matrix. Most importantly, pentagonal, 5775, and 585 topological defects were observed in the fast Fourier transformation (FFT) images (Figure 2d1, e1, and e 2) of the marked areas in Figure 2d,e, which are consistent with the results of ReaxFF MD simulation, intuitively confirming the reliability of the simulation. Furthermore, similar studies [21, 35] have shown that pentagonal and 585 defects are relatively common and stable topological defects in the carbon matrix. Therefore, the ReaxFF MD simulation and ACTEM characterization results described above are reliable.

**FIGURE 3:**
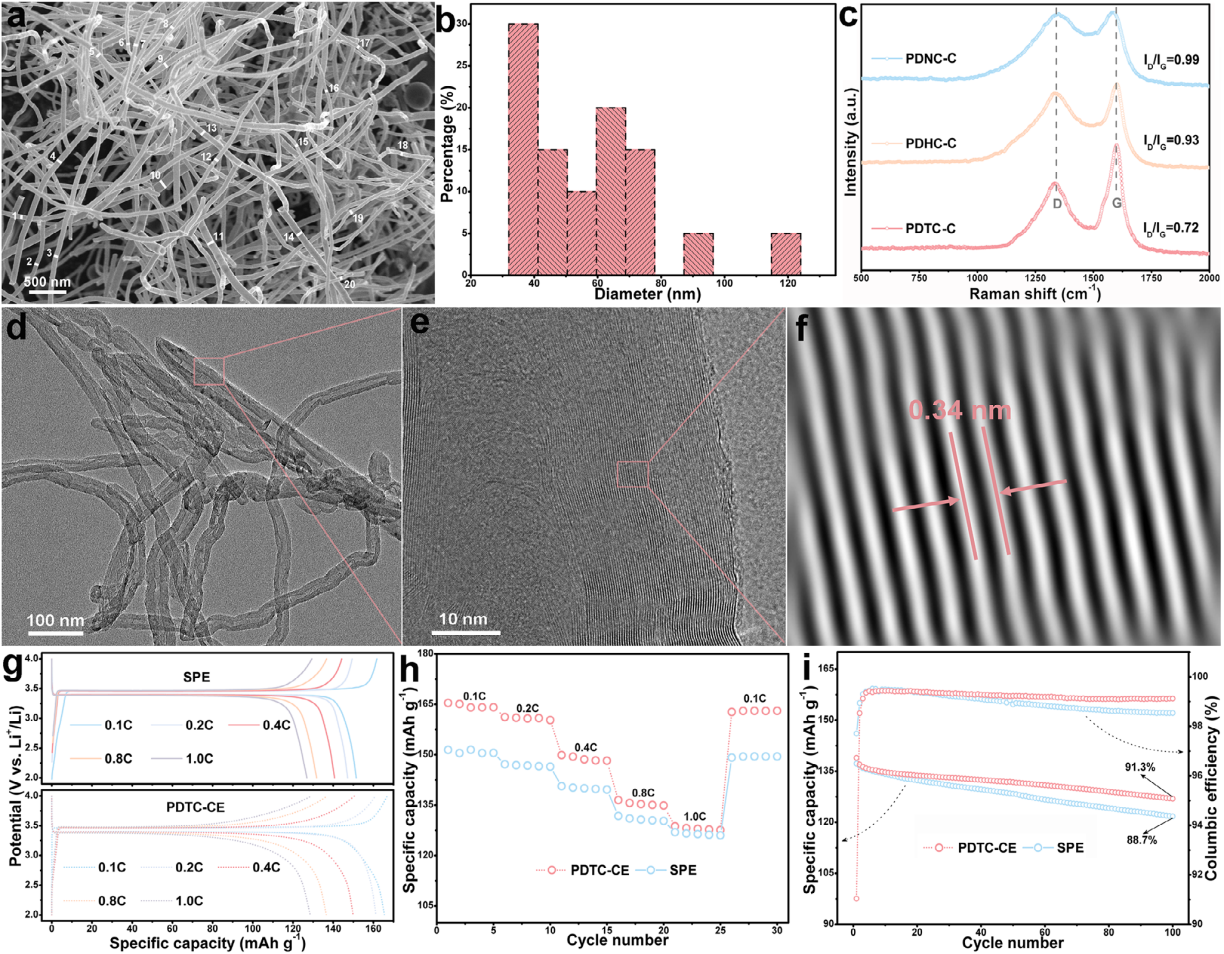
SEM (a) and diameter distribution images (b) of CNTs prepared by PDTC catalyst; Raman spectra of PDNC‐C, PDHC‐C, and PDTC‐C (c); TEM (d) and HRTEM images (e) of CNTs prepared by PDTC catalyst and magnified image (f) of the corresponding area; charge‐discharge curves at different rates of SPE and PDTC‐CE (g); rate performance image of SPE and PDTC‐CE (h); cycle stability and coulombic efficiency of SPE and PDTC‐CE at 1C (i).

After gaining a basic understanding of the topological defects in the PDTC matrix constructed by multiple JTS treatments, PDTC was used as a catalyst to study its performance in catalyzing the growth of CNTs. PDTC successfully catalytically converted acetylene carbon source into CNTs by the CVD method (confirmed by a combination of low‐ (Figure S9a) and high‐magnification (Figure 3a) SEM images), while comparative experiments showed that PDHC (Figure S9b,d) and PDNC (Figure S9c) mainly promoted the conversion of carbon source into carbon nanospheres under the same conditions. Meanwhile, the catalytic products of PDTC and PDHC reached ∼1.2 and ∼0.5 g, respectively, which reflects their mass yields of ∼1100% and ∼ 400%, respectively. Compared to PDHC, the ability to efficiently catalyze CNTs growth of PDTC is mainly attributed to its abundant topological defects based on JTS treatment. In addition, a comparative experiment using 1.36 g PDHC (which has a surface area very close to that of 0.1 g PDTC) as a catalyst showed that its catalytic products were almost entirely carbon spheres (Figure S10). This result directly indicates that the excellent catalytic performance of PDTC is not attributable to its high S_BET_, and indirectly suggests that the abundant topological defects in PDTC are the active sites that promote the catalytic growth of CNTs. The diameters of CNTs were statistically analyzed and found to be mainly concentrated in the range of 30–80 nm (Figure 3b), suggesting that these CNTs are multi‐walled CNTs. The tubular morphology of CNTs prepared by PDTC catalysis was confirmed by TEM image (Figure 3d), and the interlayer spacing of the tube wall was measured to be 0.34 nm using the FFT image of HRTEM (Figure 3e,f, and Figure S11). In addition, the element mapping of CNTs shows that its C content is as high as 97.7 at%, with only a trace amount of N and O (Figure S12). The catalytic products of all carbon catalysts were characterized by Raman spectroscopy (Figure 3c). The I_D_/I_G_ value of PDTC‐C is much smaller than that of PDNC‐C and PDHC‐C, suggesting that PDTC catalyzes the generation of highly graphitized products. In summary, all characterizations demonstrated that PDTC can be used as a catalyst to prepare CNTs, while the comparison catalysts PDNC and PDHC did not exhibit the above ability. The excellent catalytic performance of PDTC is mainly due to the abundant topological defects in its matrix, and the specific catalytic mechanism will be revealed later.

Next, the practical application value of PDTC‐C was evaluated by comparing the effects of PDTC‐C and commercial conductive agent Super‐P (SP) on the electrochemical properties of LiFePO_4_ cathode (the electrodes corresponding to PDTC‐C and SP are marked as PDTC‐CE and SPE, respectively). Figure S13a shows the cyclic voltammetry (CV) curves of PDTC‐CE and SPE at 0.1 mV s^−1^. Consistent with literature reports, they all showed a pair of basically symmetrical redox peaks in the range of about 3.3–3.6 V, which corresponds to the transformation from LiFePO_4_ to FePO_4_ during the charge–discharge process [36]. Meanwhile, the large peak intensity of the PDTC‐CE predicts its high specific capacity (SC). The rate performance of each electrode was also investigated by charge‐discharge tests at different rates (Figure 3g). The SC of PDTC‐CE and SPE decreased from ∼164.1 to ∼128.0 mAh g^−1^ and from ∼151.2 to ∼126.3 mAh g^−1^, respectively, as the discharge rate increased from 0.1C to 1C. Although the SC of PDTC‐CE decreases more significantly, its SC at each rate is greater than that of SPE, and when the rate returns to 0.1C, the SC retention rate of PDTC‐CE is slightly greater than that of SPE, reflecting the excellent cycling stability of PDTC‐CE (Figure 3h). Electrochemical impedance spectroscopy (EIS) further revealed the superiority of PDTC‐C as a conductive agent for the LiFePO_4_ cathode (Figure S13b). The intercept of the EIS curve on the real axis is attributed to the ohmic resistance (R_o_), which is the total resistance of the electrode, electrolyte, and separator. Under the same experimental conditions, it can also be considered that R_o_ mainly reflects the electrode resistance [37]. Therefore, the inset of Figure S13b intuitively shows that the electrode resistance of PDTC‐CE is smaller than that of SPE. Furthermore, the semicircle at the high frequency and the slope line at the low frequency of the EIS curve represent the charge transfer resistance (R_ct_) and Li^+^ diffusion resistance (R_L_), respectively [38]. The R_ct_ and R_L_ of PDTC‐CE are smaller than those of SPE, based on its smaller semicircle diameter at high frequency and larger slope at low frequency. The cyclic stability of PDTC‐CE was also investigated and compared with that of SPE (Figure 3i). The reversible capacity of PDTC‐CE in the first cycle is 138.9 mAh g^−1^, slightly larger than that of SPE (137.2 mAh g^−1^), and gradually decreases to 126.9 mAh g^−1^ after 100 cycles. The capacity retention rates of PDTC‐CE and SPE are 91.3% and 88.7%, respectively. At the same time, after the third cycle, the coulomb efficiency of PDTC‐CE remained above 99%, while that of SPE gradually decreased to below 99% with the increase in cycles. The above‐mentioned gratifying electrochemical performance of PDTC‐CE is mainly due to the outstanding conductivity of PDTC‐C itself and its excellent conductive network constructed based on 1D morphology.

In order to reveal the growth mechanism of CNTs using PDTC as a catalyst, the interaction between topological defects and acetylene molecules (C_2_H_2_) was first studied by DFT calculation. The graphene fragment model, including various defects, is shown in Figure S14. Pentagonal, 5757, and 585 defects cause the graphene fragments to have a certain curvature, which will be beneficial for their adsorption of C_2_H_2_. At the same time, all defects have an impact on the local charge distribution of the carbon matrix. Among them, pentagonal, 5757, and 5775 mainly affect the formation of electron‐rich sites (the sites marked with black circles in Figure S14a,b,d), while 585 and quaternary N mainly induce the formation of electron‐deficient sites (the sites marked with blue circles in Figure S14c,e).

**FIGURE 4:**
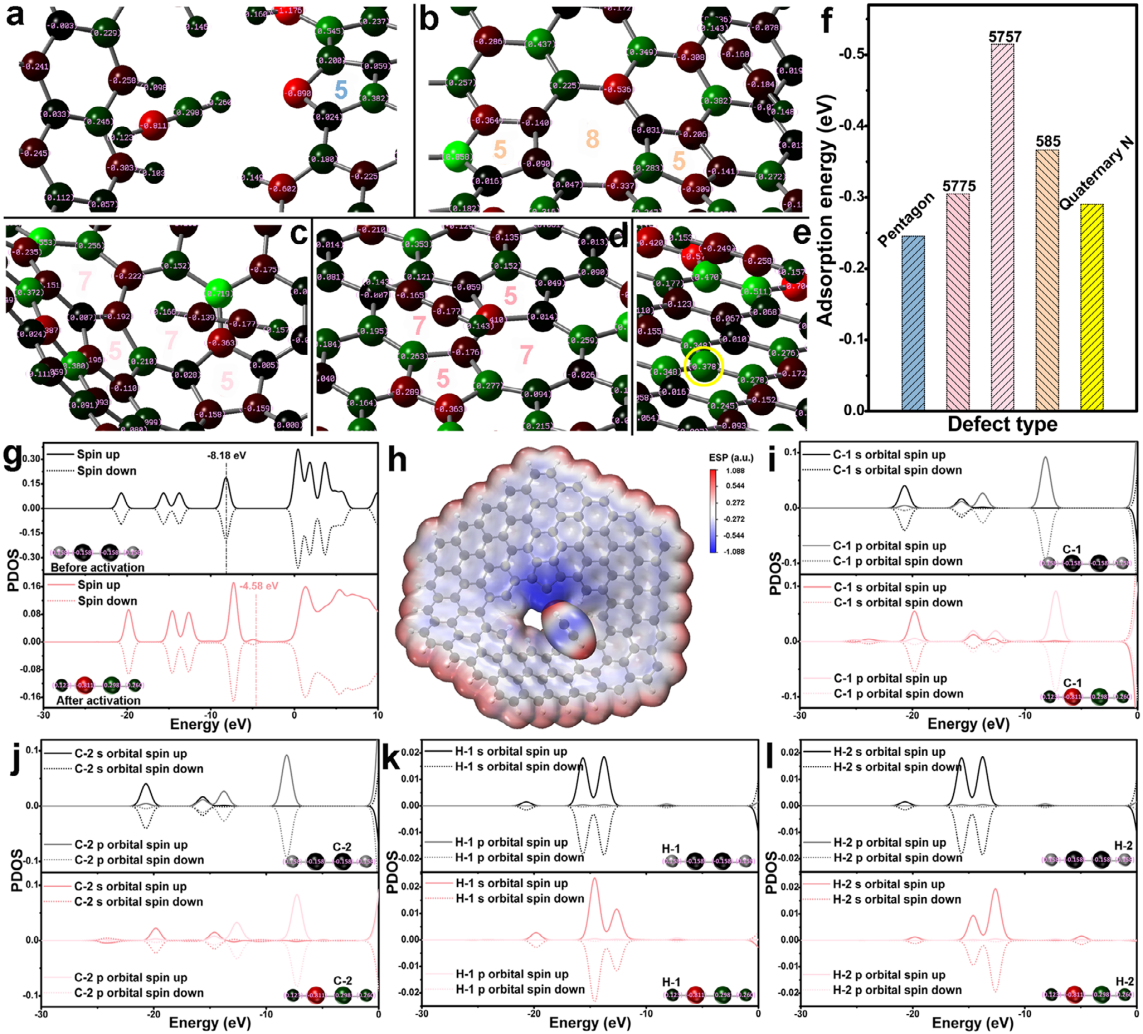
The optimal geometry of C_2_H_2_ adsorbed on pentagon defect (a), 585 defect (b), 5757 defect (c), 5775 defect (d) and quaternary N defect (e); the adsorption energy corresponding to the optimal geometric configuration of C_2_H_2_ adsorbed on various defects (f); PDOS of C_2_H_2_ before and after activation by pentagonal defect (g); electrostatic potential (ESP) map of the optimal configuration of C_2_H_2_ after activation by pentagonal defect (h); PDOS of the S and P orbitals of C‐1 (i), C‐2 (j), H‐1 (k) and H‐2 (l) atoms before and after activation of C_2_H_2_.

The optimal geometric configuration of C_2_H_2_ adsorbed on each defect and its local enlarged diagram are shown in Figure S15 and Figure 4a–e. The adsorption capacity of 5757 and 585 for C_2_H_2_ is significantly stronger than that of 5775 and quaternary N (Figure 4f), which is mainly based on their curvature structures. The above defects are mainly physical adsorption, which cannot activate C_2_H_2_ and only has a slight effect on the charge balance of C_2_H_2_ (Table S3). Fortunately, the pentagonal defect causes C_2_H_2_ to be activated by electron transfer (Figure 4a, the charge balance of C_2_H_2_ is broken, and its two carbon atoms carry positive and negative charges, respectively), and the adsorption energy between it and the activated molecule is minimal. Comparing the PDOS diagrams before and after C_2_H_2_ activation, it can be found that the band gap between HOMO and LUMO of the activated molecule is reduced, and the small peak below the Fermi level means that the activated molecule has obtained 0.25 electrons (calculated by integrating the area) from the pentagonal defect (Figure 4g). In addition, the PDOS (Figure S16) of C_2_H_2_ after interacting with other defects further illustrates that these defects have only a weak effect on the charge balance of C_2_H_2_. However, it is foreseeable that these slight effects will facilitate the further activation of C2H2 by pentagonal defects. The electrostatic potential (ESP) diagram is often used to examine the electrostatic interactions between molecules [39]. Here, the ESP diagram is used to analyze the interaction between activated C_2_H_2_ and the pentagonal defect, and the result is shown in Figure 4h. The blue and red areas represent high and low electron density, respectively. The ESP image shows that the strong blue area is located on the pentagonal defect and a carbon atom of C_2_H_2_, suggesting that they are both in a high electron density state, which also explains the phenomenon that the adsorption energy between activated C_2_H_2_ and the defect is minimal.

Furthermore, a comparative analysis was conducted on the S and P orbitals of each atom in C_2_H_2_ before and after activation by the pentagonal defect (Figure 4i–l). For unactivated C_2_H_2_, the PDOS diagrams of its two C atoms (C‐1 and C‐2, the upper part of Figure 4i,j) are exactly the same (whether S orbital or P orbital), and the same is true for its two H atoms (H‐1 and H‐2, the upper part of Figure 4k,l). For activated C_2_H_2_, the occupied electronic states near the Fermi level of each orbital of C‐1 and C‐2 (the lower part of Figure 4i,j) have changed. Combined with the results in Figure 4a, it can be found that the charge of C‐1 and C‐2 was ‐0.158 before activation, and changed to −0.811 and 0.298, respectively, after activation. The same situation happened to H‐1 and H‐2. In summary, DFT calculations indicate that the pentagonal topological defects in the carbon matrix are the main driving force for C_2_H_2_ activation. They disrupt the charge balance of C_2_H_2_ by charge transfer, while other defects only have a slight impact on the charge balance of C_2_H_2_, playing a supporting role in activation.

**FIGURE 5:**
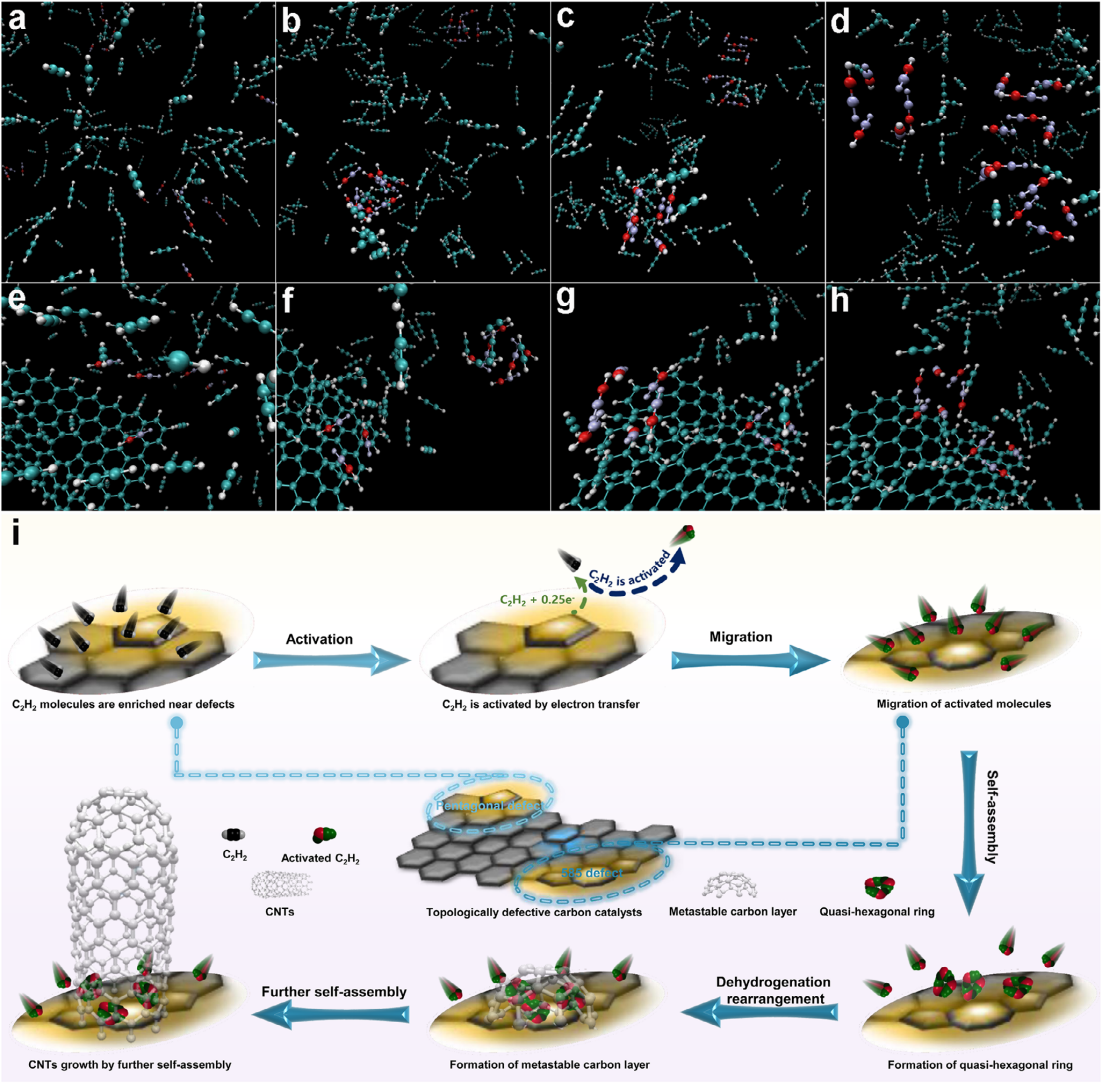
The self‐assembly process of activated molecules without the intervention of defective graphene fragments is based on MD simulations (a–d); the self‐assembly process of activated molecules mediated by a 585 topological defective graphene segment is based on MD simulations (e–h); schematic diagram of the growth mechanism of CNTs under the topological defect carbon catalysis system (i).

MD simulations have preliminarily revealed how the activated carbon source molecules form CNTs through a self‐assembly process, and the simulation results are shown in Figure 5. First, MD simulations were performed on the activated carbon source molecules without the intervention of topological defect graphene fragments (Figure 5a–d). Before the simulation begins, all activated molecules (AMs) are evenly distributed in the MD box, and some randomly distributed AMs are marked by color differences (Figure 5a). At the beginning of the simulation, Ams almost immediately (within tens of picoseconds) aggregated in varying numbers and broke the initial uniform distribution (Figure 5b). For the labeled AMs, it was intuitively observed that they belonged to two agglomeration camps. As the simulation progressed, the labeled AMs gradually connected end‐to‐end in their respective aggregation camps (possibly based on electrostatic interactions) and tended to form a quasi‐six‐membered ring structure (Figure 5c). At the end of the simulation, the quasi‐six‐membered ring structures in the two aggregation camps gradually approached each other, which was conducive to the formation of a metastable carbon structure (Figure 5d).

Subsequently, the effects of graphene fragments containing various defects on the AMs self‐assembly process were studied based on MD simulations (Figure 5e–h, and Figure S17). For 585 defects, the simulation has not started, all AMs are still evenly distributed in the MD box, and several AMs are marked by different colors (Figure 5e). Similar to the simulation process without defective graphene fragments, the marked AMs immediately divided into two aggregation camps at the beginning of the simulation, one of which was on the surface of the defective graphene fragment and the other was far away from the defective graphene fragment (Figure 5f). As the simulation continued, the AMs in each aggregation camp also connected end to end to form a quasi‐six‐membered ring structure (Figure 5g). Moreover, the quasi‐six‐membered ring structures in the two aggregation camps gradually approached each other, and by the end of the simulation, they were very close (Figure 5h). It is particularly noteworthy that the aggregation camp on the surface of the 585 defective graphene fragments immediately formed the quasi‐six‐membered ring structure at the beginning of the simulation, and this structure remained until the end of the simulation. Combined with quasi‐six‐membered rings from another aggregation camp that is very close to this structure, it will be possible to form a metastable carbon layer on the surface of 585 defective graphene fragments. Similar to the 585 defective graphene fragment, quasi‐six‐membered ring structures also formed on the surface of the 5775 defective graphene fragment (Figure S17b), suggesting the potential formation of metastable carbon layers on its surface. However, for the remaining defects, no quasi‐six‐membered ring structures were observed on the surface of their graphene fragments (Figure S17a,c,d), meaning that these defects may not affect the self‐assembly process of AMs.

Regarding the growth mechanism of CNTs, the vapor‐liquid‐solid (VLS) theory was initially accepted by researchers under the catalytic system of metal nanoparticles [40, 41]. However, later on, the growth of CNTs was also observed under oxide catalysis [42] and even pure carbon catalysis [43]. Obviously, the VLS theory does not apply to these new catalyst systems. Therefore, the vapor‐solid‐solid (VSS) theory was proposed. This theory holds that the carbon sources are activated on the surface of the catalyst to form intermediates (decomposed into free radicals or activated); then, these intermediates form metastable carbon layers at the curvature sites by free radical condensation or self‐assembly combined with dehydrogenation rearrangement; finally, the edge‐active metastable carbon layers continue to form CNTs through further self‐assembly [44]. In this study, C_2_H_2_ was used as the carbon source, which is extremely difficult to decompose into free radicals due to its high C≡C bond energy (837 kJ/mol). Fortunately, the above simulation calculations bring two key insights: first, C_2_H_2_ is activated by disrupting its charge balance through electron transfer based on pentagonal topological defects, and the remaining defects play a minor auxiliary role in the activation of C_2_H_2_ by slightly affecting its charge balance; second, the activated molecules tend to form a quasi‐hexagonal ring structure by connecting end to end on the surface of the topological defect with a certain curvature. Combining these key insights with the existing VSS theory, the growth process of CNTs in this study is proposed as follows: first, C_2_H_2_ is activated by electron transfer from pentagonal topological defect and the slight assistance of other defects; then, activated molecules migrate to the surface of topological defects with a certain curvature and self‐assemble through electrostatic interaction to form a quasi‐hexagonal ring structure, and further form a metastable carbon layer by dehydrogenation rearrangement; finally, the metastable carbon layer with edge activity continuously self‐assembles to form CNTs according to the defect curvature (Figure 5i). Characterization of the time‐sliced catalytic samples indirectly confirmed this growth process: the presence of metastable carbon layer synapses (marked by red circles) was observed in the sample about 5 min after the start of catalysis (Figure S18a); meanwhile, some newly emerged tubular substances were discovered in the sample corresponding to about 20 min of the catalytic reaction (Figure S18b, marked by red circles), and an open tube‐end structure was also confirmed (Figure S18c). In short, by combining simulation calculations with existing theories, a possible growth mechanism for CNTs applicable to this study is proposed, which involves the synergistic interaction of multiple topological defects. Although the growth mechanism requires further in‐depth analysis, the existing research results have provided new insights into the identification of active centers for the growth of CNTs catalyzed by special carbons and how this special catalytic growth process proceeds.

## 3 Conclusion

In summary, this study demonstrates the feasibility and efficiency of constructing topological defects in a carbon matrix by JTS technology. Meanwhile, topological defect carbon prepared by JTS technology is used as a catalyst to grow CNTs through the CVD process. The research results show that this unique catalytic system can not only achieve the successful preparation of CNTs, but also apply the catalytic product as a conductive agent in lithium‐ion batteries, whose performance is even slightly better than commercial conductive agent. Research on the growth mechanism of CNTs under this special catalytic system found that the carbon source molecules are activated through electron transfer from pentagonal topological defects and the slight assistance of other defects; then, the activated molecules formed a metastable carbon layer by self‐assembly combined with dehydrogenation rearrangement on the topological defect surface with a certain curvature; finally, the carbon layer grew into CNTs through further self‐assembly based on its edge activity. Consequently, this study demonstrated the high efficiency of JTS technology in constructing topological defects in the carbon matrix, and that the topological defect carbon can catalyze the growth of CNTs, and further proposed a reasonable growth mechanism.

## Conflicts of Interest

The authors declare no conflicts of interest.

## Supporting information




**Supporting File**: advs73542‐sup‐0001‐SuppMat.docx.

## Data Availability

The data that support the findings of this study are available from the corresponding author upon reasonable request.
