# Supplementary material for: Topological Defects in Carbon Matrix are Efficiently Constructed by Joule Thermal Shock for Catalyzing the Growth of Carbon Nanotubes
Source: Adv Sci (Weinh). 2026 Jan 9;13(12):e20422. doi: 10.1002/advs.202520422 (PMC12948207; doi:10.1002/advs.202520422)
Supplement: Supplementary file 1 — Supporting File: advs73542‐sup‐0001‐SuppMat.docx. [file ADVS-13-e20422-s001.docx]

**Topological defects in carbon matrix are efficiently constructed by Joule thermal shock for catalyzing the growth of carbon nanotubes**

Bin Wang ^a, *^, Shizhen Dong ^b^, Guoan Xie ^b^, Yanling Yu ^c^, Nuoxin Wang ^d^, Huakun Liu ^e^, Xian Jian ^f^, Jinyang Li ^b^, Zuowan Zhou ^b^, Jianhui Qiu ^a, *^

a School of materials science and engineering, Xihua University, Chengdu 610031, Sichuan, P. R. China.

b School of Chemistry, Southwest Jiaotong University, Chengdu 610031, Sichuan, P. R. China.

c School of Chemistry and Chemistry Engineering, Harbin Institute of Technology, Harbin 150001, Heilongjiang, P. R. China.

d Key Laboratory of Cell Engineering of Guizhou Province, Affiliated Hospital of Zunyi Medical University, Zunyi 563003, Guizhou, P. R. China.

e Institute of Energy Materials Science (IEMS), University of Shanghai for Science and Technology, Shanghai 200093, P. R. China.

f School of Materials and Energy, University of Electronic Science and Technology of China, Chengdu, 611731, P. R. China.

Corresponding author. E-mail address: jeasonbin@163.com (B. Wang), qiu@akita-pu.ac.jp (J. Qiu).

**Abstract:** This study aims to establish a simple and efficient method for preparing topological defect carbon (TDC) and reveal its catalytic mechanism for the growth of carbon nanotubes (CNTs). TDC is prepared using N-doped carbon as a precursor through multiple Joule thermal shock treatments, and the efficiency of this method in constructing topological defects in the carbon matrix is confirmed by multi-dimensional characterization. Subsequently, the TDC catalyzed the growth of CNTs, and a possible catalytic growth mechanism based on the synergistic interaction of multiple defects is proposed. The mechanism proposes that the carbon source molecules are activated through electron transfer from pentagonal topological defects and the slight assistance of other defects, and then the activated molecules form a metastable carbon layer by self-assemble combined with dehydrogenation rearrangement according to the defect curvature, which further self-assembles to achieve the growth of CNTs based on the edge activity of the carbon layer. Therefore, this study not only brings new perspectives to clarify the growth mechanism of specialty carbon-catalyzed CNTs, but also provides an efficient research and development platform for this type of specialty carbon catalysts.

**Experimental**

**Materials.** Pyrrole, ammonium persulfate (APS), and concentrated hydrochloric acid (HCl) were of analytical grade and purchased from Aladdin Biochemical Technology Co., Ltd. Ar and C_2_H_2_ were provided by Chengdu Honghaoyuan Gas Co., Ltd. Pyrrole was purified before use, and the other reagents did not require pretreatment.

**Preparation of topological defect carbon catalysts.** First, the polypyrrole (PPy) precursor was prepared as follows: concentrated HCl (10.5 mL) and pyrrole monomer (0.5 mL) were added to deionized water (50 mL) and stirred at room temperature for 10 min; then, APS solution (0.83 M, 10 mL) was added dropwise to the above mixture, and the reaction was stirred at room temperature for 1 h; finally, the product was washed and dried at 60 ℃ for 12 h to obtain the PPy precursor.

The PPy precursor was pre-carbonized at 800 ℃ for 1 h in an Ar atmosphere, and PPy-derived N-doped carbon (PDNC) was obtained. Subsequently, the PDNC was subjected to multiple JTS treatment to prepare topological defect carbon catalysts (PDTC). The JTS equipment (ZKJY-HTS) was purchased from Shenzhen Zhongke Jingyan Technology Co., Ltd., and the specific JTS process was as follows: 80 mg PDNC was placed between two pieces of graphite paper (thickness is 0.05 mm, working area is 1 × 2.5 cm^2^), heat treated for 3000 ms at a discharge voltage of 32 V and a pulse current of 75 A in an Ar environment, then cooled for 1000 ms, and the single JTS process was repeated 50 times (Fig. S1, the highest treatment temperature reached 1225 ℃). After the above JTS process, PDTC was prepared. At the same time, in order to compare the efficiency difference between JTS technology and conventional high-temperature heteroatom removal method for constructing topological defects, PDNC was also heat-treated in Ar atmosphere at 1200 ℃ for 1 h, and a comparison sample PDHC was prepared.

**Evaluation of catalyst performance.** The catalytic growth of CNTs was carried out by CVD method using PDTC as catalyst and C_2_H_2_ as carbon source gas. In a typical catalytic experiment, PDTC (0.1 g) was placed in a CVD furnace and Ar (100 sccm) was introduced for 1 h to exclude the air, and then the temperature was raised to 850 ℃ (5 ℃ min^-1^) in a mixed atmosphere of C_2_H_2_ and Ar (50 sccm : 50 sccm) for 2 h. For comparison, PDNC and PDHC were used as catalysts and underwent the above CVD process under the same experimental conditions, respectively. In addition, a comparative experiment was designed to verify that the excellent catalytic performance of PDTC is not mainly based on its high S_BET_. The comparative experiment repeated the above catalytic process using 1.36 g of PDHC as catalyst (according to the nitrogen adsorption-desorption isotherm test, 1.36 g PDHC has a surface area that is very close to that of 0.1 g PDTC).

Meanwhile, the catalytic product was used as a conductive agent for lithium-ion batteries, and its performance was compared with that of the commercially available conductive agent SP. A coin-type half-cell (CR2032) was assembled with lithium metal as the counter electrode, Celgard 2400 as the separator, and LiPF_6_ solution (1 M in a 1:1 v/v dimethyl carbonate and ethylene carbonate mixture) as the electrolyte. The working electrode was prepared with a mass ratio of LiFePO_4_, conductive agent (catalytic product or commercial Super-P) and binder (polyvinylidene fluoride, PVDF) of 8:1:1. The slurry was evenly coated on aluminum foil and vacuum-dried at 100 ℃ for 12 h, and then cut into small pieces with a diameter of 12 mm to obtain the working electrode (with a mass loading of 2 ~ 3 mg cm^-2^). All the above materials are purchased from Shenzhen Kejing Zhida Technology Co., Ltd. Galvanostatic measurements were performed at 25 ℃ on Neware Test System between 2.0 and 4.0 V at different rates. The cyclic voltammetry (CV) and electrochemical impedance spectroscopy (EIS) were performed by a CHI 660E electrochemical workstation.

**Characterization.** The morphology of the samples was observed by scanning electron microscopy (SEM, HITACHI S-4800) and transmission electron microscopy (TEM, TECNAI F30). The elemental composition and chemical state of each element of the samples were analyzed by X-ray photoelectron spectroscopy (XPS, carried by a PHI Quantum 5000 equipped with an Al Kα source), and the spectra were calibrated by the C1s peak (284.6 eV). The defect structures of the samples were revealed by Raman spectroscopy (HORIBA Xplora with excitation laser beam wavelength of 532 nm) and fine near-edge X-ray absorption spectra (NEXAFS, performed at beamline 20A1 of Taiwan Light Source of National Synchrotron Radiation Research Center). Furthermore, thermogravimetric analysis (TGA, NETZSCH STA449 F3, it was carried out in the experimental temperature range at a ramp setting of 10 ℃ min^-1^ from room temperature to 800 ℃ in air) of PDTC and PDHC was performed to indirectly demonstrate the extremely high defect density of PDTC. Meanwhile, the aberration-corrected TEM (ACTEM) image of PDTC was obtained by a double aberration-corrected Titan Themis Z electron microscope at an accelerating voltage of 80 kV. In addition, the N_2_ adsorption-desorption isotherms of the samples were also tested by an ASAP-2020 Physisorption Analyzer.

**Physicochemical simulation and calculation.** In this work, classic molecular dynamics (MD) simulations are performed to investigate the structural transformations of curved N-doped graphene sheet (simulation model) subjected to JTS process from molecular level. To this end, a MD box with dimensions of around 50 × 51 × 80 Å^3^ is created, in which N-doped graphene sheet composed of 1568 C atoms, 128 N atoms (the atomic ratio among pyridinic N, pyrrolic N and graphitic N is 1:1:2) and 192 H atoms is placed. Periodic boundary conditions (PBCs) are imposed in the three orthogonal directions to mimic large sample. To describe the atomic interactions in the N-doped graphene-based systems, the reactive forcefield (ReaxFF) potential is utilized. Prior to MD simulations, as-generated sample is optimized to a local configuration with energy and force tolerances of 1.0 × 10^-4^ Kcal/moL and 1.0 × 10^-4^ Kcal/moL Å, respectively. Soon afterwards, MD simulations are performed to further relax the sample with 100,000 timesteps at temperature of 300 K under NVT (constant number of particles, constant volume, and constant temperature) ensemble, in which the temperature is controlled by Nose-hoover thermostat. Finally, as-relaxed samples are thermally annealed radiated with 50 cycles under NVT ensemble (40,000,000 timesteps), with temperature varying from 773/4173 ~ 4173/773 K. The atom motions in the diamond-based systems follow the classical Newton’s motion, in which the velocity-Verlet algorithm with timestep of 0.1 fs is applied to integrate the classic Newton’s equation. All the MD calculations are implemented using the Large-scale Atomic-Molecular Massively Parallel Simulator (LAMMPS) software package. ^[45]^

Based on the understanding of the topological defect structure in the PDTC matrix through MD simulation and ACTEM characterization, the interaction between the topological defect active sites and the C_2_H_2_ carbon source was further revealed by density functional theory (DFT) calculations. GaussView 5.0 was used to select the cluster model for topological defect structure modeling (the graphene fragment containing the defect structure has about 100 C atoms, and the edge area is saturated with H atoms). Here, the periodic graphene model was not selected for modeling because this model cannot reflect the deformation and wrinkles of graphene caused by defective structures. Molecular structure optimization and single-point energy calculation were performed using Gaussian 16 software. ^[46]^ The functional and basis set used in the structure optimization process are UB3LYP/TZVP, and the functional and basis set used in the single-point energy calculation of the optimized structure are UB3LYP/6-311+G**. In addition, the GD3BJ method is used to correct the dispersion force during the calculation process, thereby improving the description accuracy of the weak interaction force. The partial density of state (PDOS) of C_2_H_2_ and its composite systems with different defective carbon models were analyzed by wave function processing software Multiwfn 3.8, ^[47]^ and the Hirshfeld method was used to extract the PDOS data of C_2_H_2_. Furthermore, the Mulliken method was used to extract the PDOS data of the s and p orbitals of the four atoms (2 C atoms and 2 H atoms) of C_2_H_2_. The electron density and electrostatic potential (ESP) of the optimized structure were still calculated using Multiwfn 3.8 softwave, ^[47]^ and the calculation results were loaded into the visualization program VMD, ^[48]^ which can map the difference in ESP to the isosurface of the electron density in different colors.

In order to understand how the activated molecules achieve the growth of CNTs based on the self-assembly process, further MD simulations were performed. In this simulation, a MD box with a size of 50 × 50 × 50 Å^3^ was constructed, which contained 200 activated molecules, or a mixed system of 1 defective graphene fragment and 200 activated molecules. All simulations were performed using GROMACS v2018.8^[49]^ software using non-periodic boundary conditions. The GAFF force field^[50]^ was used to describe the interactions between activated molecules, and the force constants of defective graphene fragments were calculated based on the Hessian matrix using the Sobtop program. ^[51]^ In addition, the atomic charges of the activated molecules and defective graphene fragments were obtained by the aforementioned DFT calculations. The specific simulation details are as follows: the conjugated gradient method (CG) is used to minimize the energy of the system; the equilibrium stage is carried out in the NVT ensemble (the number of atoms, volume and temperature are all constant), the temperature is maintained at 298.15 K, and the simulation duration is 5 ns; Newton’s equations of motion are solved by integration using the Leap-frog algorithm with a time step of 2 fs; and the temperature is controlled by a velocity-rescale heat bath. Moreover, van der Waals force and electrostatic interactions are considered simulthaneously in the simulation, and non-bonded interactions between all particles are calculated at each step.

**Joule thermal shock device.**


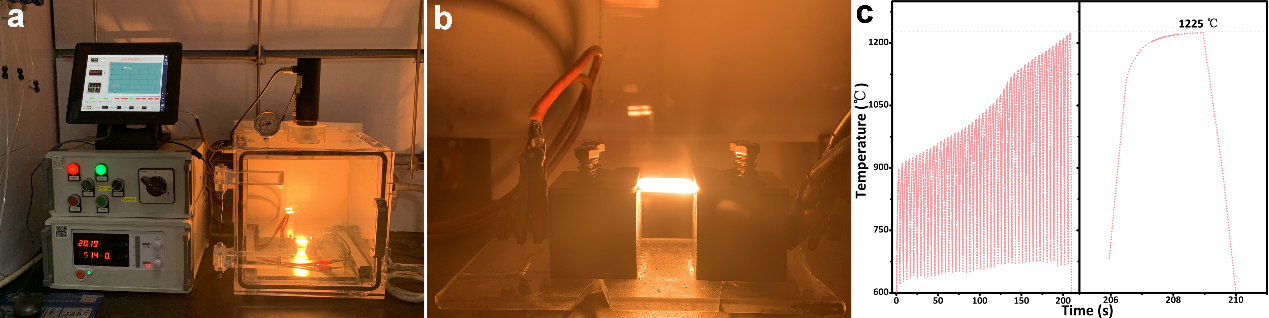


**Fig. S1.** Actual picture of the Joule thermal shock device (a, b) and its temperature curve during operation (c).

**Structure characterization, simulation calculation and performance testing**


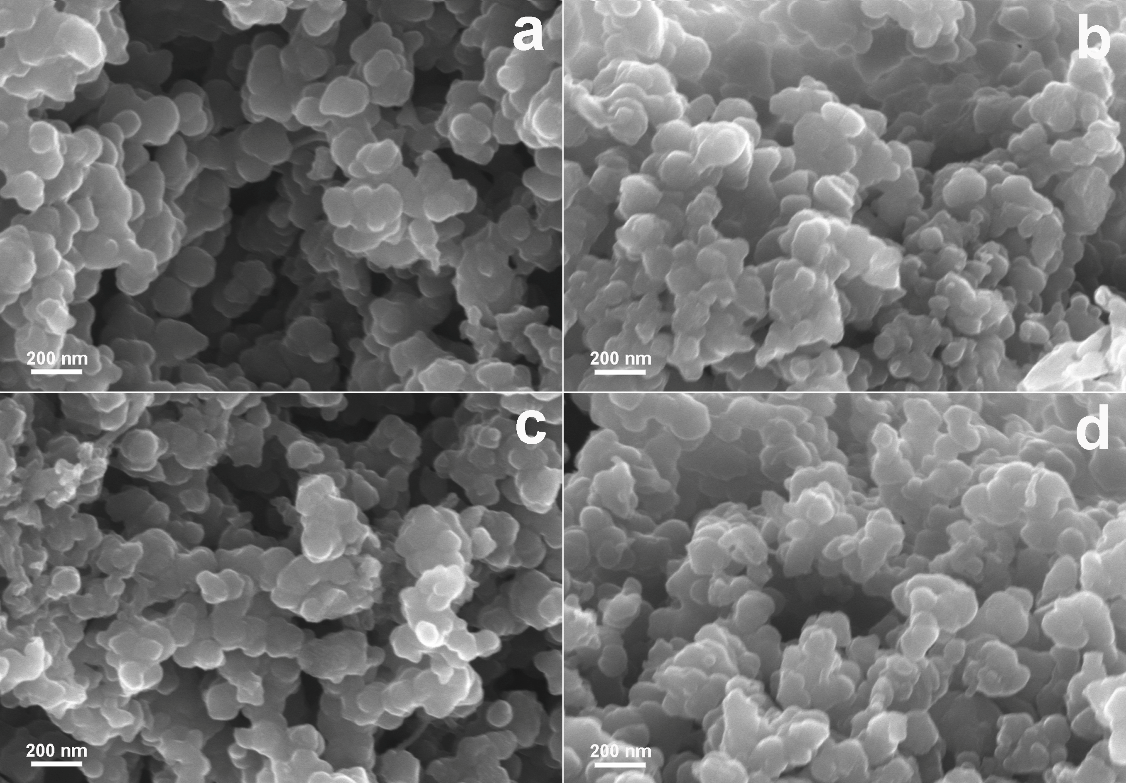


**Fig. S2.** SEM images of PPy (a), PDNC (b), PDTC (c) and PDHC (d).


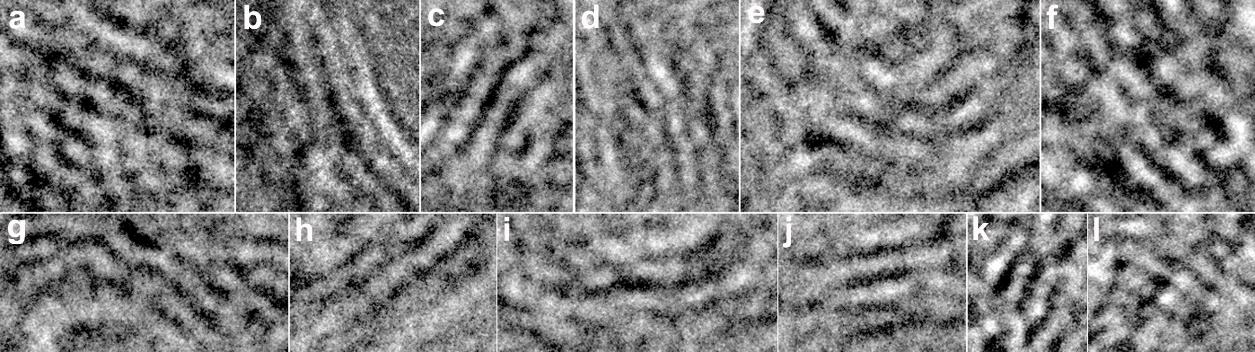


**Fig. S3.** The graphite microcrystalline structures in PDHC.


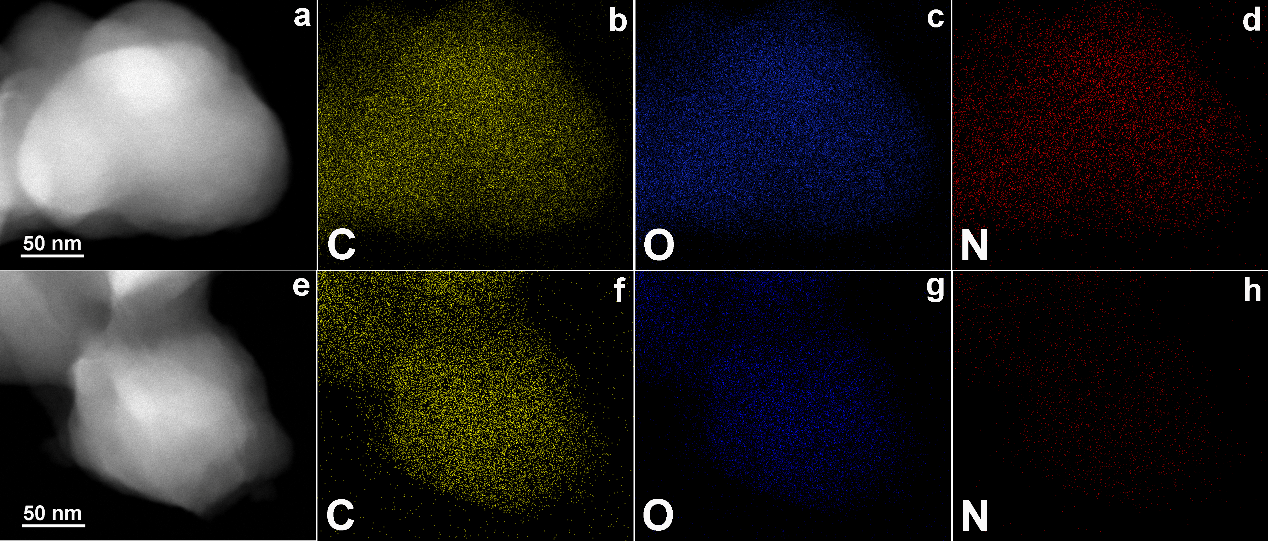


**Fig. S4.** Dark-field TEM, C, O and N elemental mapping STEM images of PDNC (a ~ d) and PDHC (e ~ h).

**Table S1.** Chemical composition of the samples

| Sample | Element mapping analysis of TEM | | |  | XPS spectra analysis | | |
| --- | --- | --- | --- | --- | --- | --- | --- |
|  | C/at% | O/at% | N/at% |  | C/at% | O/at% | N/at% |
| PPy | --- | --- | --- |  | 72.30 | 15.40 | 12.30 |
| PDNC | 85.83 | 5.89 | 8.28 |  | 85.00 | 5.80 | 9.20 |
| PDHC | 94.41 | 4.06 | 1.53 |  | 93.40 | 4.30 | 2.30 |
| PDTC | 93.56 | 4.32 | 2.12 |  | 91.80 | 5.20 | 3.00 |


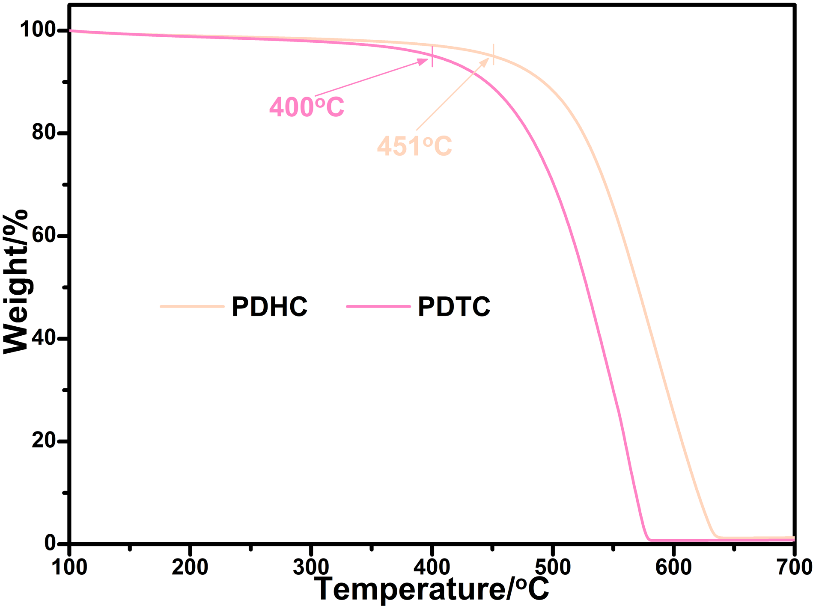


Fig. S5 TG curve of PDTC and PDHC under air.


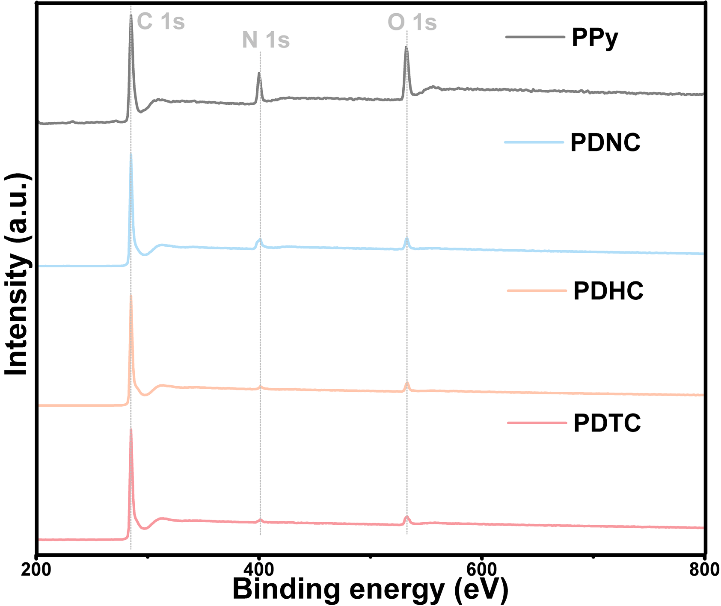


**Fig. S6.** XPS survey spectra of PPy, PDNC, PDHC and PDTC.


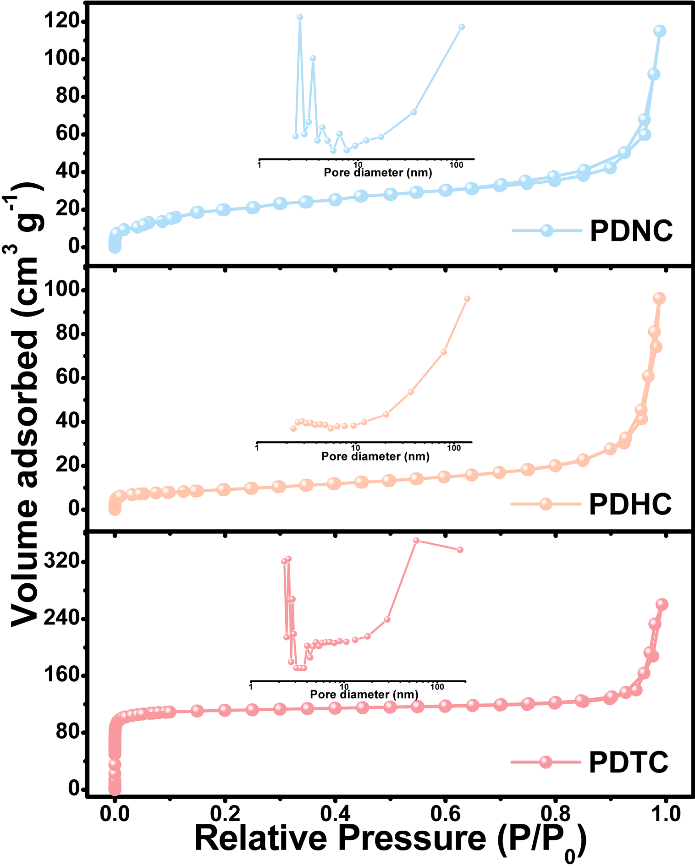


**Fig. S7.** Nitrogen adsorption/desorption isotherm and pore size distribution of PDNC, PDHC and PDTC.

**Table S2.** The pore structure parameters of the samples

| Sample | N_2_ adsorption-desorption analysis | | | |
| --- | --- | --- | --- | --- |
|  | S_BET_ (m^2^ g^-1^) | V_T_ (cm^3^ g^-1^) | D_av_ (nm) | V_mic_ (cm^3^ g^-1^) |
| PDNC | 98.79 | 0.17 | 3.62 | 0.02 |
| PDHC | 32.39 | 0.14 | 9.24 | 0.01 |
| PDTC | 443.70 | 0.40 | 1.82 | 0.16 |

D_av_: average pore diameter; V_T_: total pore volume; V_mic_: micropores volume


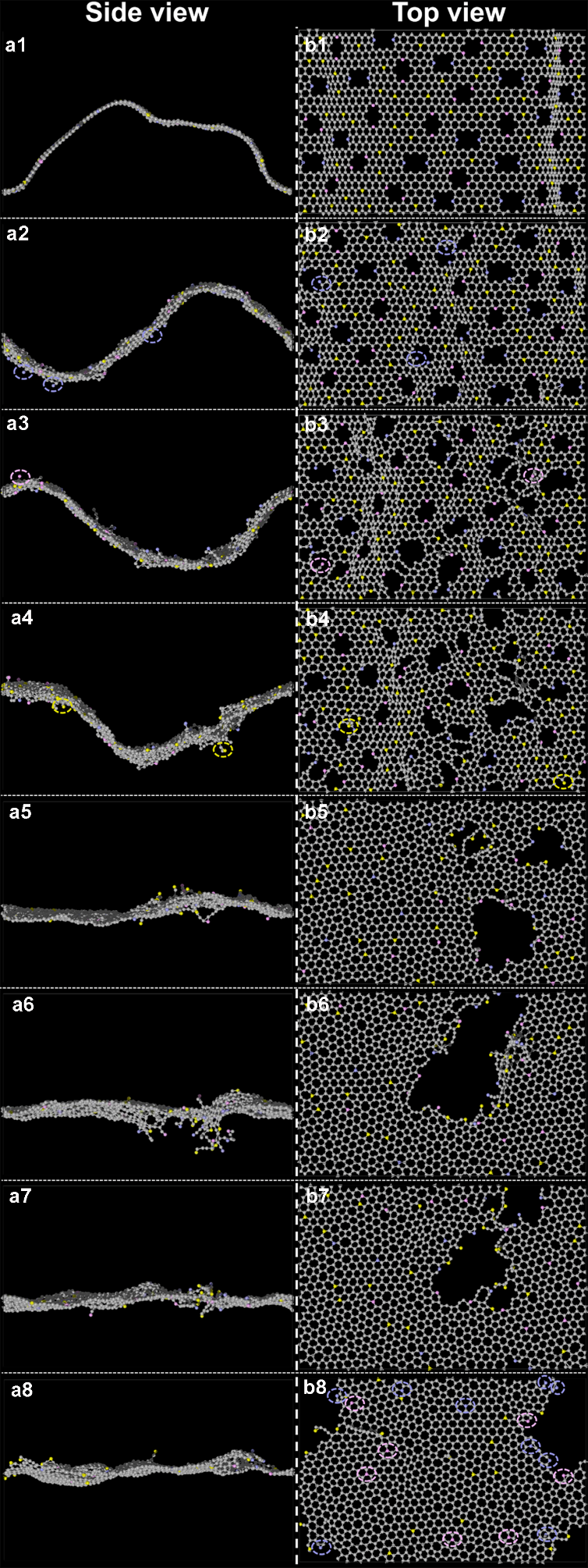


**Fig. S8.** Side view and top view snapshots of the model in a molecular dynamics calculation.


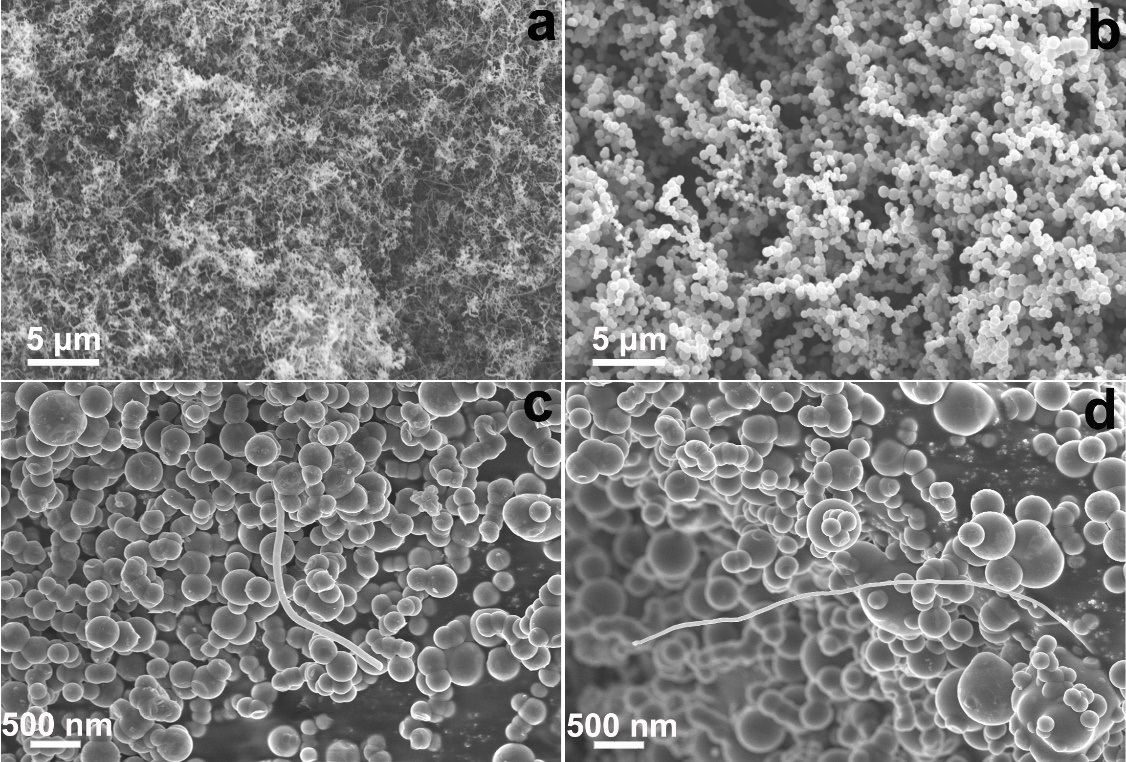


**Fig. S9.** SEM images of the catalytic products of PDTC (a) PDHC (b, d) and PDNC (c).


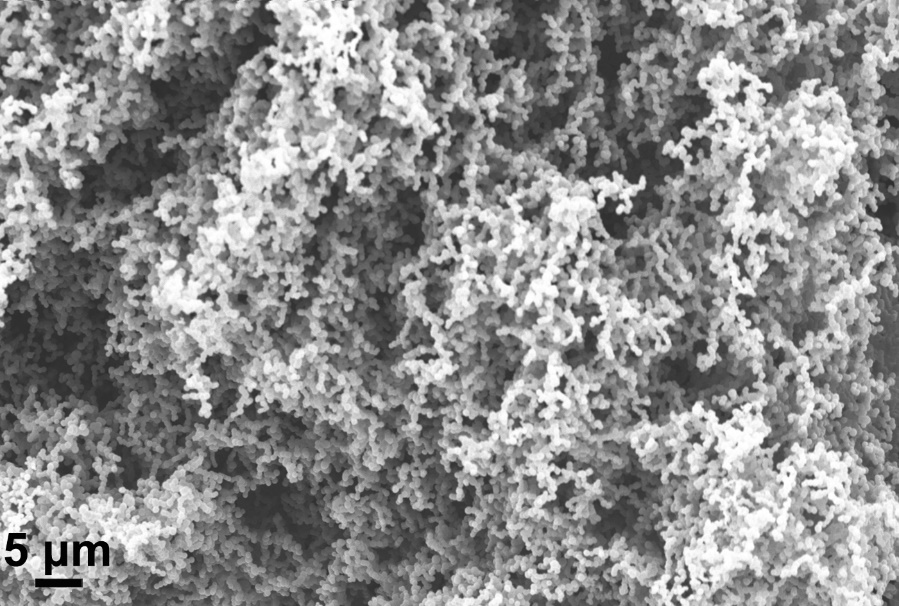


Fig. S10 SEM image of the catalytic products of 1.36 g PDHC.


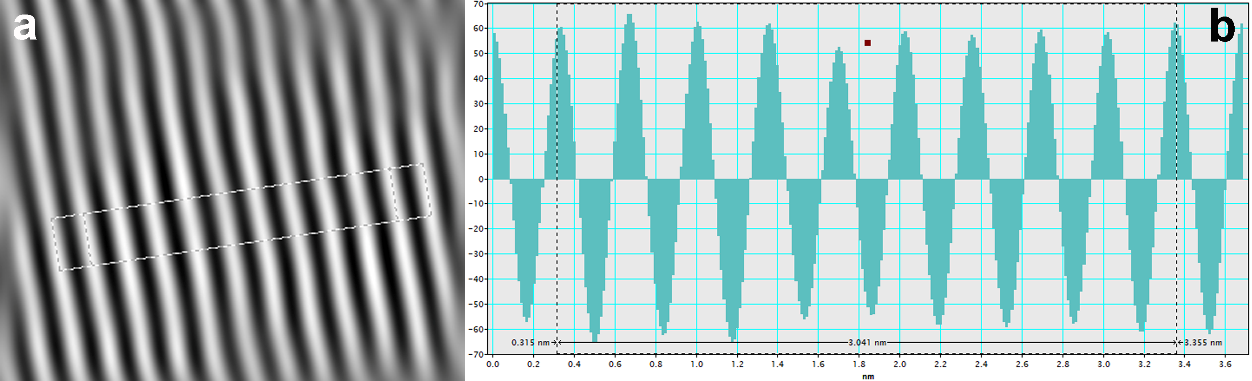


**Fig. S11.** HRTEM magnified image of the tube wall of CNTs prepared by PDTC catalysis (a), and the measurement image of the graphite layer spacing of the tube wall (b).


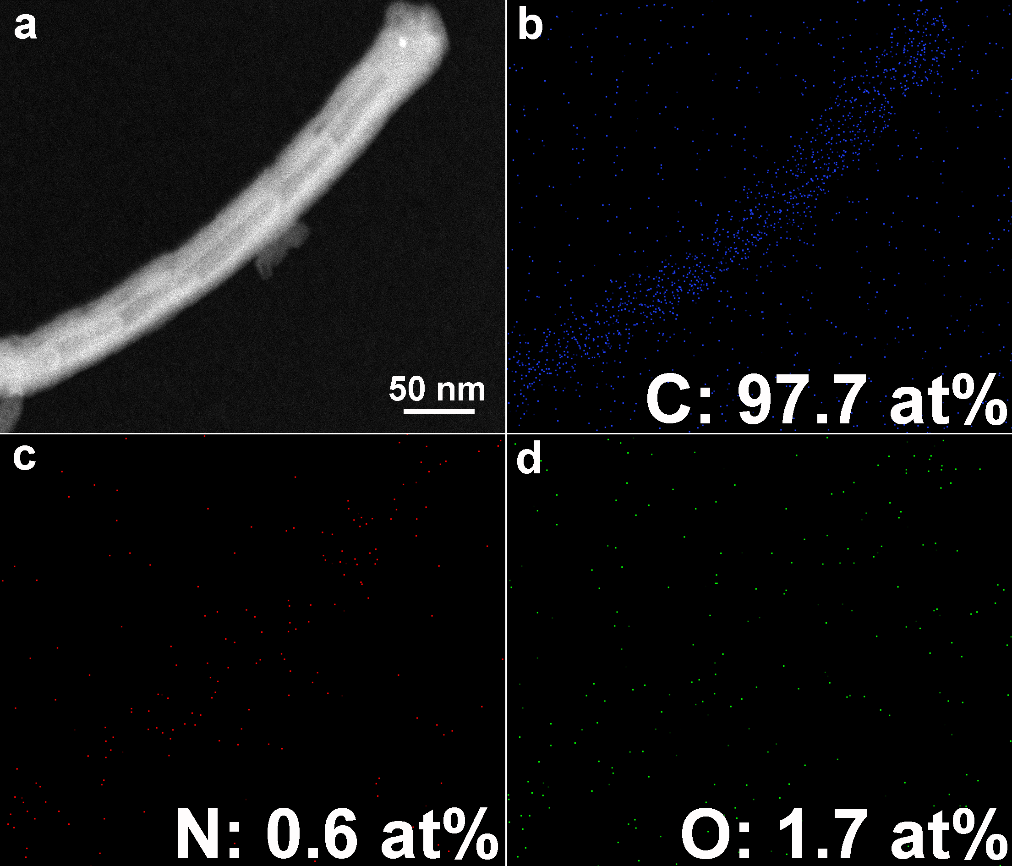


**Fig. S12.** Dark-field TEM, C, O and N elemental mapping STEM images of CNTs prepared by PDTC catalysis.


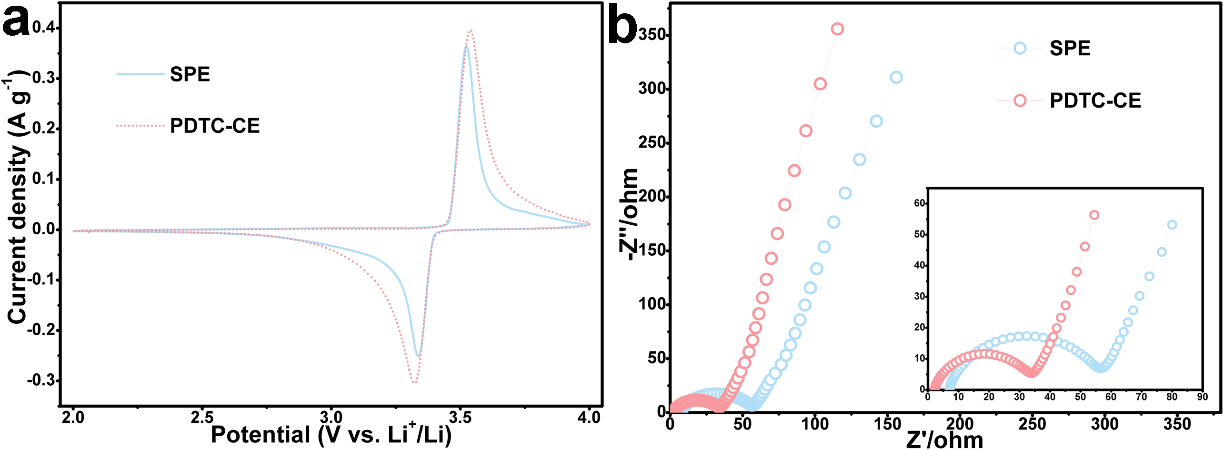


**Fig. S13.** CV (a, at 0.1 mV S^-1^) and EIS (b) curves of SPE and PDTC-CE.


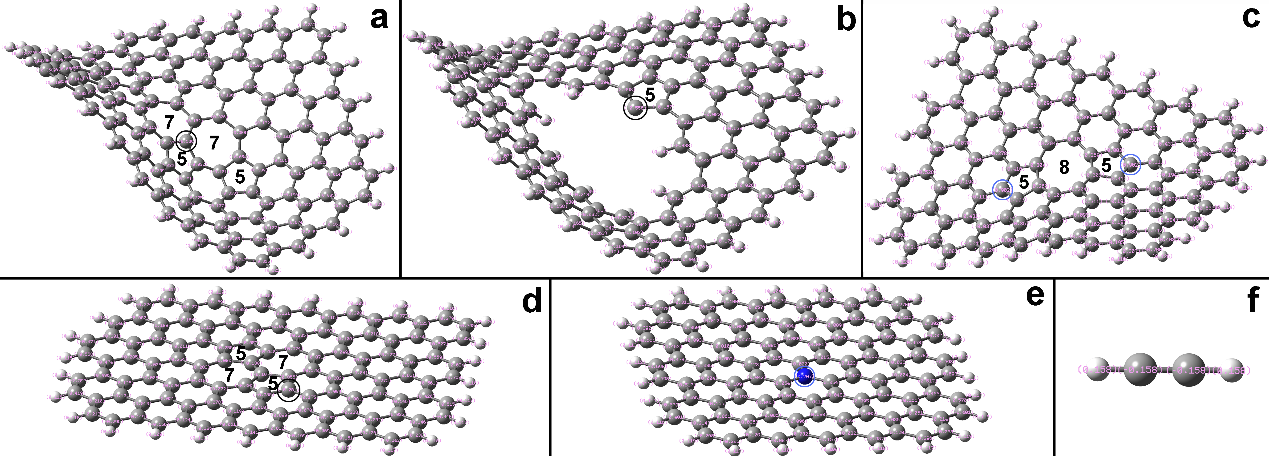


**Fig. S14.** Structure models of 5757 defect (a), pentagon defect (b), 585 defect (c), 5775 defect (d), quaternary N defect (e) and C_2_H_2_ molecular (f).


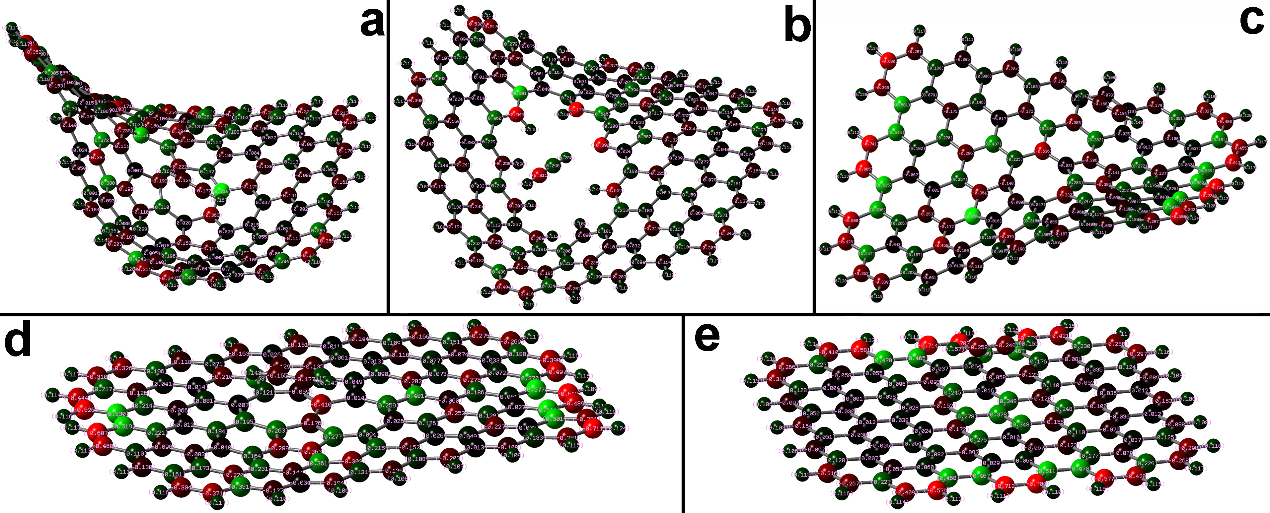


**Fig. S15.** The optimal geometry of C_2_H_2_ adsorbed on 5757 defect (a), pentagon defect (b), 585 defect (c), 5775 defect (d) and quaternary N defect (e).

**Table S3.** The charge of the carbon atoms in a C_2_H_2_ molecule after the C_2_H_2_ molecule interacts with various defects

| Models | C1 | C2 |
| --- | --- | --- |
| C_2_H_2_ | -0.158 | -0.158 |
| C_2_H_2_ with pentagon | -0.811 | 0.298 |
| C_2_H_2_ with 585 | -0.168 | -0.184 |
| C_2_H_2_ with 5775 | -0.165 | -0.177 |
| C_2_H_2_ with 5757 | -0.139 | -0.177 |
| C_2_H_2_ with quaternary N | -0.249 | -0.258 |


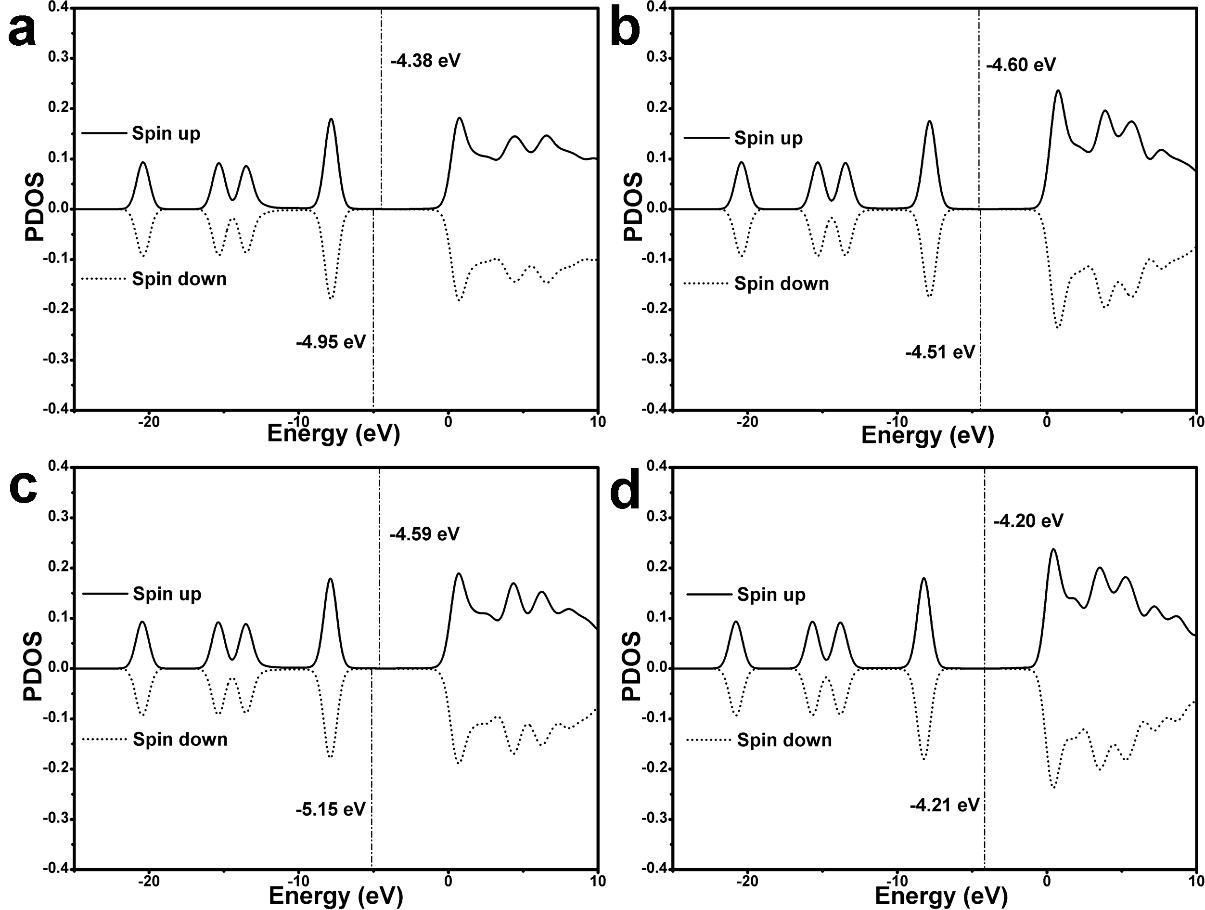


**Fig. S16.** PDOS of C_2_H_2_ after it was affected by 5757 defect (a), 5775 defect (b), 585 defect (c) and quaternary N defect (d).


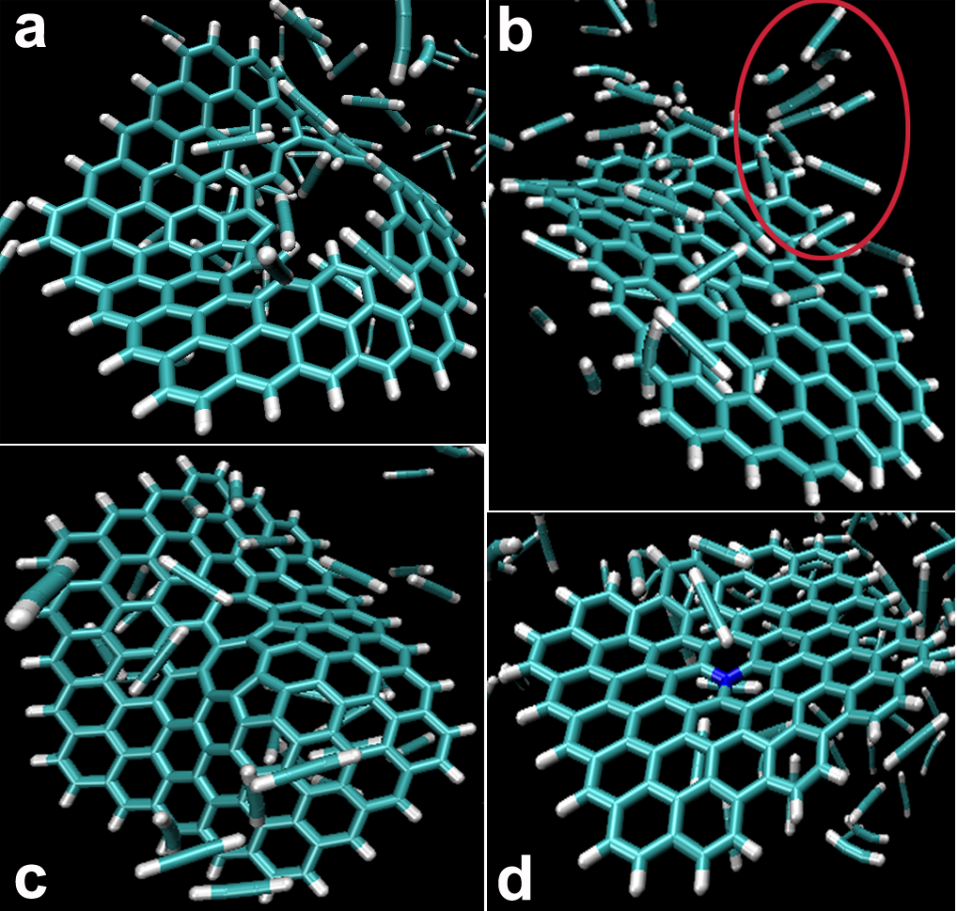


**Fig. S17.** MD simulation results of activated molecules self-assembly mediated by pentagon defect (a), 5775 defect (b), 5757 defect (c) and quaternary N defect (d).


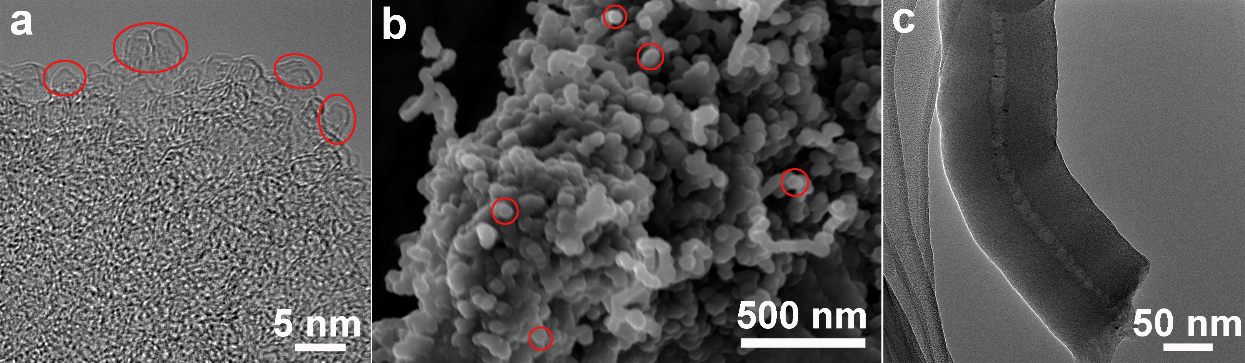


**Fig. S18.** HRTEM image of the sample catalyzed by PDTC for about 5 min; SEM and TEM images of the sample catalyzed by PDTC for about 20 min.
